# Examining acute psychopharmacological effects of nicotine vaping versus heated tobacco products in a randomised crossover study of product naïve adult smokers

**DOI:** 10.1038/s41598-023-49602-3

**Published:** 2023-12-19

**Authors:** Dimitra Kale, Harry Tattan-Birch, Jamie Brown, Sharon Cox, Lynne Dawkins, Maciej L. Goniewicz, Kierra Morris, Lion Shahab

**Affiliations:** 1 Department of Behavioural Science and Health, University College London, London, UK; 2 SPECTRUM Consortium, London, UK; 3 Centre for Addictive Behaviours Research, School of Applied Sciences, London South Bank University, London, UK; 4 Department of Health Behavior, Roswell Park Cancer Institute, Buffalo, NY USA

**Keywords:** Psychology, Human behaviour

## Abstract

Nicotine vaping products (NVPs) and heated tobacco products (HTPs) are designed to replicate the sensory and behavioural aspects of smoking cigarettes while avoiding combustion. The success of these products as harm reduction tools will partially depend on their ability to satisfy smokers and alleviate nicotine-related withdrawal symptoms. This study aims to compare short-term effects of NVPs (Juul and Aspire PockeX) versus HTPs (IQOS) on smoking-related withdrawal relief, product satisfaction, intention to switch to NVP/HTP, perceptions and attitudes in UK adult cigarette smokers naïve to these products. In a randomized cross-over study, 45 participants visited the lab twice, at each visit trying one of the two products (NVP/HTP) and completing a questionnaire. Responses were normalized on a 0–100% scale and mean differences (MD) between NVP and HTP scores computed, with positive and negative MD values indicating greater endorsement for NVP and HTP, respectively. Cigarette cravings were reduced similarly (~ 20.0%) by both products (MD = 4.5%, 95%Confidence Interval (CI) − 4.8, 13.8). Direct positive effects (MD = − 3.5%, 95%CI − 7.2, 0.2) and adverse side effects (MD = 1.8%, 95%CI − 0.3, 3.8) were comparable after each product use, though marginally favouring HTPs. HTPs were perceived as more satisfying overall (MD = − 13.2%, 95%CI − 20.3 − 6.1) than NVPs but both were perceived as similarly addictive (MD = 3.6%, 95%CI − 4.6, 11.8), relative to cigarettes. Intention to switch to either product was comparable (MD = 4.0%, 95%CI − 5.7, 13.8). Comparison of acute use of NVP versus HTP in a sample of UK smokers naïve to these products suggests that HTPs are perceived as more satisfying than NVPs, though still less satisfying than cigarettes.

*Registration*: The analysis plan was pre-registered, and it is available at https://osf.io/6ymdu

## Introduction

Combustible cigarette (hereafter cigarette/s) smoking remains one of the leading causes of preventable disease and death worldwide, directly responsible for ~ 8 million global deaths each year^1^. Most smokers want to quit^2^ and many try to quit each year^3^, but the chances of success of any given quit attempt are low, with fewer than 5% of unaided attempts succeeding for 12 months or more^4^. This is because smoking behaviour is primarily maintained by psychopharmacological addiction to nicotine delivered rapidly by cigarettes^5^. Even when smokers switch to nicotine replacement therapy, chances of success are modest at 12-months, around 8%^6,7^. Importantly, cigarette addiction also involves sensorimotor cues and behavioural rituals associated with smoking (i.e., the act of lighting and puffing a cigarette, the sensation of smoke in the airways, and the taste and smell of the cigarette), which smokers need to be overcome during quitting^8^. Over the last decade, new products, such as nicotine vaping products (NVPs) and heated tobacco products (HTPs), have entered the market that deliver nicotine without combustion, while closely mimicking aspects of the sensorimotor and behavioural components of cigarette smoking. These products may enable harm reduction, i.e., the replacement of a more harmful with a less harmful product, for those smokers who cannot or do not want to stop using nicotine completely^9,10^. Currently the adult NVP and HTP use prevalence in England is 9.3% and 0.2% respectively^11^. The majority of NVP users are ex- or current smokers but 30.1% of current smokers have never used an NVP in Great Britain^12^, and ever use of HTP is only 0.4% in England^11^.

NVPs (also known as e-cigarettes and vapes), are battery operated devices that aerosolize liquids that usually contain a mixture of glycerol and propylene glycol, flavours, and nicotine. Evidence shows NVPs are less harmful to health than cigarette smoking since users are exposed to much lower levels of toxicants and carcinogens^13,14^, and switching from cigarette smoking to NVP use is associated with improvements in respiratory symptoms such as asthma and chronic obstructive pulmonary disease and lower risk of cardiovascular diseases^9,15,16^. Randomised controlled trials^13,17,18^ and observational studies^19,20^ also suggest that NVPs can increase the likelihood that people will succeed in their attempts to stop smoking cigarettes, roughly doubling abstinence rates compared with nicotine replacement therapy (NRT), nicotine-free electronic cigarettes, no support or behavioural support only. NVPs have also replaced medicinal NRT as the preferred aid for smokers making a quit attempt in England^19,21^.

HTPs are an expanding category within the novel tobacco product marketplace. They differ from cigarettes in that they aim to heat rather than burn tobacco, and from NVPs, because they use tobacco rather than nicotine e-liquid to generate an aerosol. By heating tobacco instead of burning it, HTPs could substantially reduce the number of harmful substances that arise from burning tobacco according to research by the tobacco industry (i.e.,^22,23^). Emerging evidence suggests that HTPs are likely more harmful than NVPs, with some estimates ranging from 1.5 to 2 times more harmful than NVPs^24,25^. On the other hand, a recent study reported comparable harmful effects of NVPs and HTPs on endothelial cell migration^26^. There is currently little evidence on their effectiveness in replacing cigarette smoking in any country^10,25,27^ apart from Japan and Italy^28,29^. In Japan one observational study suggests that the introduction of HTPs led to a reduction in cigarette sales^28^, while in Italy a randomised control trial comparing the effectiveness of HTPs and NVPs on cigarette substitution found that switching to HTPs elicited a reduction in cigarette consumption which was comparable to NVPs^29^. It will take some time to undertake expensive, long-term trials to evaluate the effectiveness of HTPs in cigarette reduction and smoking cessation. Though, since NVPs are effective in cigarette reduction and smoking cessation (i.e.,^13,17^), if HTPs have a similar psychopharmacological profile to NVPs after acute use, this would increase confidence that like NVPs, HTPs could be a harm reduction tool.

The extent to which these products will be used by cigarette smokers and contribute to harm reduction depends on several factors but at least partially on how effective they are in controlling craving and in providing cigarette-related withdrawal relief as well as general satisfaction. Acute NVP use appears to reduce the urge to smoke and withdrawal symptoms^30–33^. However, these reductions are greater in smokers experienced in NVP use^32^ than in smokers naïve to NVPs^30,31,33^, but not always as great as those seen following cigarette smoking^30,33^. From the limited research available on HTPs, use may reduce cravings from cigarette more effectively than NVPs among smokers naïve to these products^34^, though—again—this reduction may not be as great as after cigarette smoking^35^. NVP use is also associated with very few side effects and high levels of satisfaction and enjoyable sensations throughout the respiratory track (i.e., throat, tongue, windpipe, nose, and lung) in experienced users^32,36,37^. Similarly, experienced HTP users report few side effects, while throat satisfaction, the sensation felt in the throat during the first seconds after taking a puff, is weaker than from cigarette smoking^38^. Qualitative research exploring facilitators and barriers to using NVPs and HTPs in smokers naïve to these products suggests that there is an interest and curiosity in uptake and use of both products, and NVPs are seen as easier to use than HTPs^39^. Additionally, throat discomfort (i.e., throat sensations of harshness) and high anticipated cost as well as lack of health knowledge related to NVPs and HTPs were identified as barriers to the use of both products^39^.

Given these results and the potential harm reduction role of NVPs and HTPs, it is important to evaluate how NVPs and HTPs impact withdrawal symptoms related to cigarette smoking of cigarette smokers naïve to these products. Such evaluation could inform intervention design and public health messaging to promote cigarette smoking cessation and switching to potential harm-reducing nicotine delivery devices. In particular, there is limited independent research directly comparing acute use of NVPs versus HTPs on psychopharmacological outcomes in cigarette smokers naïve to either product^10,27,40^. If HTPs are comparable to NVPs, this would increase confidence that like NVPs, HTPs could be a helpful harm reduction tool. Thus, the aim of the present study is to compare short-term effects of NVP versus HTP use among cigarette smokers naïve to these products in (i) cigarette smoking related withdrawal relief, (ii) product satisfaction, (iii) intention to switch to NVPs and HTPs, (iv) perceptions about NVPs and HTPs compared with cigarettes, (v) attitudes towards NVPs and HTPs, and (vi) reasons for motivating smokers to switch from cigarettes to NVPs and HPTs.

## Methods

### Design

This randomised cross-over study (AB/BA design) forms part of a wider study assessing the possible health effects of using novel nicotine and tobacco-containing products compared with smoking cigarettes and not smoking at all. The current analysis focuses on determining differences in cigarette-related withdrawal relief, product satisfaction, and risk perceptions for NVPs compared with HTPs among cigarette smokers naïve to using these products. Two questionnaires were administered to participants (before and after product use) at each of two laboratory visits at University College London, lasting approximately 1 h each. The wider study also involved the collection of biological samples, not reported here. Participants were reimbursed for time and travel.

### Sample and recruitment

Participants were recruited in the Greater London (UK) area between March 2018 and February 2022, with a pause between March 2020 and September 2021 due to the COVID-19 pandemic, using various recruitment methods to access a diverse sample in terms of sociodemographic characteristics (age and sex). These included adverts in newspapers, social media, online NVP forums, approaching users on the street, email mailouts, as well as use of marketing companies.

Participants were screened for eligibility via an online questionnaire. Inclusion criteria for the current study required participants to have smoked an average of five or more cigarettes per day for at least six months and not have regularly used NVPs or HTPs in their lifetime. Current smoking status was verified using a breathalyser to assess expired air carbon-monoxide (CO); readings above 8 ppm indicated current smoking. Due to collection of biological samples (as part of the wider study but not reported here), participants were excluded if they were younger than 18 years old, had a history of heart or lung disease, were pregnant, or had bleeding gums, illness, or infection within 24 h of their scheduled appointment. Forty-five participants were recruited, and this sample provided 90% power to detect medium effects (f = 0.25) for within group analyses. The power calculation was based on detecting effects for biological outcomes (not presented here) in cross-sectional analysis.

The study received ethical approval from the University College London Ethics Committee (Project ID 12621/001). All methods were performed in accordance with the relevant guidelines and regulations. Informed consent was obtained from all subjects.

### Procedures

Participants visited the lab twice and were asked to refrain from smoking, eating food, drinking alcohol, or using the lavatory from one hour before each lab visit. After taking consent, participants completed a baseline questionnaire to assess socio-demographics, smoking-related characteristics and psychopharmacological outcomes. They then provided breath and urine samples for toxicological analysis. Following sample collection, participants were walked to an outside area and asked to use either a NVP or an HTP, provided by the study team, for 5 min. No specific instructions were given to participants about how to use the product in these 5 min, apart from how to switch it on and off. After product use, participants completed a follow-up questionnaire to assess psychopharmacological outcomes. At the second visit, participants again completed a baseline questionnaire on psychopharmacological outcomes, tried the other product, and completed a follow-up questionnaire to assess psychopharmacological outcomes after product use. The order of product use was randomised to account for sequence effects. Excel random number generator function was used to randomise the participants to which product they used first and which one second.

### Materials

The NVP: The ‘Aspire PockeX’ or the ‘Juul’ were randomly assigned to each participant (20 participants have been randomised to Juul and 25 to Aspire PockeX; unequal numbers were due to a delay in obtaining Juul). The ‘‘Aspire PockeX’ is an all-in-one second-generation NVP kit with a 1500mAh internal battery and integrated 2 ml e-liquid tank. Participants could choose an e-liquid with 18 mg/ml concentration of nicotine, with tobacco, fruit or menthol flavour. The Juul is a slim NVP that has no buttons or switches and uses a regulated temperature control. It is charged through USB ports and has prefilled cartridges (‘pods’) of 18 mg/ml nicotine concentration in UK and participants could choose tobacco, mint, mango or menthol flavour. These NVPs were selected on the basis of nicotine delivery efficiency (Juul) and popularity (Aspire PockeX) in UK when the study begun^41,42^. NVPs were fully charged prior to the session.

The HTP: ‘IQOS’ is an HTP manufactured by Philip Morris, which heats specially designed tobacco sticks called HEETS. IQOS was the most popular HTP in the UK when the study begun. HTPs were fully charged prior to the session and participants could choose tobacco smooth, rich tobacco, or menthol HEETS of 18 mg/g nicotine to use. The amount of nicotine per gram of tobacco in a regular IQOS tobacco stick is ~ 15.4 mg/g and in a menthol tobacco stick is ~ 16.4 mg/g^27^. Evidence suggests that IQOS provide less efficient nicotine delivery than cigarettes and Juul, but more than Aspire PockeX^43^. While NVPs provide lower peak nicotine levels and lower overall nicotine levels to users than cigarette smoking^9^.

### Measures

Socio-demographics included age (continuous), sex (male vs other), ethnicity (white vs other), and education (post-16 qualification vs not).

Smoking-related characteristics included length of smoking (in years), cigarette dependence (measured with five questions of Fagerstrom test of cigarette dependence^44^; (i) number of cigarettes smoked per day (ii) How soon after you wake up do you smoke your first cigarette? (iii) Do you find it difficult to stop smoking in no-smoking areas? (iv) Which cigarette would you hate most to give up? (v) Do you smoke more frequently in the first hours after waking than during the rest of the day? (measured continuously). Motivation to stop (Motivation to stop scale (MTTS)^45^, continuous), and past 12 months quit attempts (yes/no).

### Outcomes

Supplementary Table 1 lists details of the outcomes, including response options.

(i) Withdrawal symptoms related to cigarettes were assessed before and after use of the NVP/HTP product. We used a question derived by the Mood and Physical Symptoms Scale (MPSS^46,47^) which assesses momentarily (right now) cigarette craving strength. We also assessed six other general mood and physical symptoms related to withdrawal symptoms before and after use of the NVP/HTP product. The scale examining mood and physical symptoms includes an item related to poor sleep, which was excluded from the present analysis since participants did not get a chance to sleep after using each product.
(ii) Product satisfaction:
  - For assessing acute positive effects of NVP/HTP after their use, 14 items based on previous research^48^, such as ‘hit’, ‘satisfaction’, ‘feels like smoking’ were assessed. We calculated an overall positive effect score by summing all items, but we also reported responses to individual items.
  - To assess adverse side-effects of NVP/HTP immediately after their use, 21 items based on previous study^33^, such as ‘confused’, ‘dizzy’, ‘headache’ were assessed. An adverse side-effect score was calculated summing all items, but we also reported responses on the individual items.
(iii) Intention to switch to NVP/HTP
  Participants were asked to rank how likely they were to switch to NVP after NVP use, and how likely they were to switch to HTP after HTP use, on a 4-point Likert scale (0 = ‘very unlikely’ to 3 = ‘very likely’).
(iv) Perceptions about NVPs/HTPs compared with cigarettes
  Participants were asked after product use compared with cigarettes (i) whether they think the NVP/HTP is safe, (ii) whether the NVP/HTP is addictive, and (iii) how satisfying is NVP/HTP. People could respond “don’t know” to the first two above questions. In the main analysis ‘don’t know’ answers were excluded. Sensitivity analysis included individuals with ‘don’t know’ responses and this option was placed in between the most popular response options of each product to have the least biasing impact on results.
(v) Attitudes
  Participants were asked after product use (i) whether they found the product helpful in enabling them to refrain from smoking, (ii) how pleasant they found the product to use, (iii) how embarrassing they found the product to use in the company of others, (iv) whether they would recommend the product to a friend who wanted to stop smoking, (v) whether they would use the product outdoors, (vi) indoors at home, (vii) indoors at work/public spaces.
(vi) Reasons that would motivate switching from cigarettes to NVPs/HPTs
  Participants were asked to indicate the importance of the following nine reasons in motivating them to switch from cigarettes to NVP/HTP after using the products. If the product (i) ’is satisfying’, (ii) ‘is less smelly (than cigarettes)’, (iii) ‘is being used by friend(s)’, (iv) ‘helps to manage stress’, (v) ‘is less harmful than cigarettes’, (vi) ‘is less harmful to other people around’, (vii) ‘can be used in smoke-free areas’, (viii) ‘can help to stop smoking’, (ix) ‘can help to cut down smoking’.

All responses were normalised on 0% to 100% scale, with higher values indicating higher endorsement of the items, and all measures have been used in previous research^49^.

### Statistical analysis

Descriptive statistics were calculated to characterise the sample. For all analyses below, we report the mean difference (MD) in scores for NVPs versus HTPs alongside 95% confidence intervals (CI), with positive MD values indicating greater endorsement for NVPs and negative values greater endorsement for HTPs.

Linear regressions were conducted to examine the differences in (i) cigarette-related withdrawal symptoms after using NVP and HTP, adjusted for urges or mood and physical symptoms (respectively) before using each product. Similarly, to examine differences between NVP and HTP in (ii) product satisfaction, (iii) intention to switch to NVP/HTP, (iv) perception, (v) attitudes, (vi) reasons for switching to NVP/HTP, linear regressions were conducted. The intercept/constant of these regressions were the estimate, and 95% CIs were calculated by multiplying the standard error appropriate critical value from the t-distribution. All the regressions were adjusted for period.

Statistical analysis was conducted on complete cases in SPSS Statistics version 27. The protocol and analysis plan were pre-registered on Open Science Framework (https://osf.io/6ymdu).

An exploratory analysis examined potential differences between the two NVPs used (Juul and Aspire PockeX) in product satisfaction and throat irritation. We examined this because prior studies suggest that the aerosol from NVPs that use nicotine salts e-liquid, such as Juul, is less harsh to inhale than aerosol from products using freebase nicotine, like the Aspire PockeX^50^.

**Table 1:** Socio-demographic and smoking-related characteristics.

	Total (N = 45)
Socio-demographics	
Mean age (SD)	35.7 (15.0)
% Male (N)	60.0 (27)
% White (N)	66.7 (30)
% Post-16 qualification (N)	57.8 (26)
Smoking-related characteristics	
Mean length of smoking, years (SD)	15.7 (13.4)
Mean cigarettes dependence (SD)	2.8 (2.2)
Mean age of starting smoking (SD)	19.7 (6.2)
Mean motivation to stop smoking (SD)	3.5 (1.6)
% Any past 12 months quit attempts (N)	31.1 (14)

SD = standard deviation. Cigarette dependence was measured with the Fagerstrom test of cigarette dependence, scores 1–9 with higher scores presenting higher cigarette dependence. Motivation to stop was measured with the motivation to stop scale, scores 1–7 with higher score presenting higher motivation to quit cigarette smoking.

### Ethics approval

The study has been approved by UCL Research Ethics Committee (Project ID Number: 12621/001). All methods were performed in accordance with the relevant guidelines and regulations. Informed consent was obtained from all subjects.

## Results

Table 1 provides demographic and smoking related characteristics of the 45 participants. Their mean age was 36 years, with slightly more men than women included, just over half had post-16 qualifications and two thirds were of white ethnicity. On average, participants started smoking at age 20, had smoked for an average of 16 years, and reported relatively low cigarette dependence. A third of participants had reported a quit attempt in the past 12 months, and participants reported moderate motivation to quit smoking.

**Figure 1:**
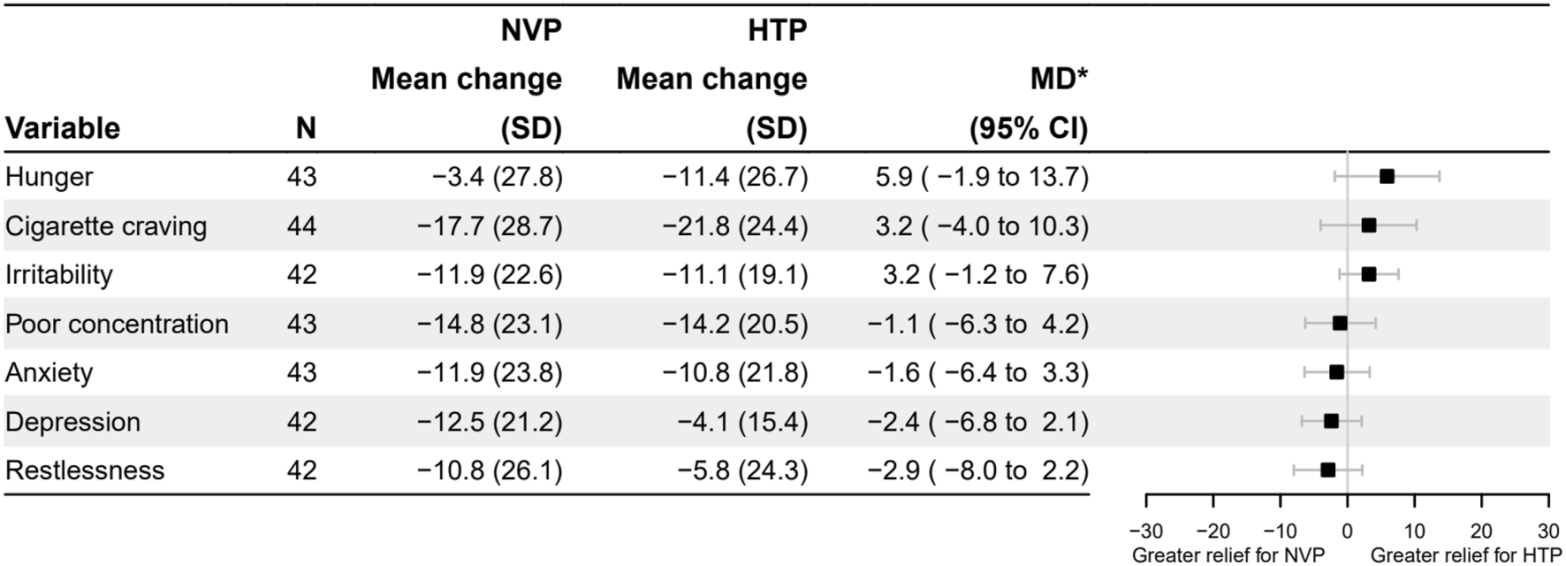
Cigarette smoking related withdrawal relief after NVP and HTP use. NVP = nicotine vaping product, HTP = heated tobacco product, SD = standard deviation, MD* = mean within-person difference in the outcome variable after NVP use minus HTP use with adjustment for both period and the mean difference in variable before product use, CI = Confidence Interval. Cigarette cravings were measured on a 6-point Likert scale from 1 = ‘no urges’ to 6 = ‘extremely strong’. All the others were measured on a 5-point Likert scale, 1 = ‘not at all’ to 5 = ‘extremely’. All scales were normalised on 0% to 100%.
(ii) Product satisfaction
  - The overall acute positive effects score was close to the middle value of the scale for both products, and slightly more favourable after HTPs than after NVPs use, though differences were non-significant (Fig. 2). Overall adverse side-effects score was very low after product use, but slightly less favourable after NVPs than HTPs use, though again this was not significant (Fig. 3). Examining the individual acute positive effect scores following use, participants reported that the HTPs reduced their craving for nicotine more than the NVPs, that it was more satisfying, and that it felt more like their usual brand. In addition, participants reported a greater burning sensation and throat hit from the NVPs than HTPs (Fig. 2).

(i) Cigarette-related withdrawal relief
  Product use reduced withdrawal symptoms related to cigarettes, with no difference in the reduction between products (i.e., mean scores of cigarettes craving strength: before NVP use M = 43.1%, SD = 18.1, after NVP use M = 25.5%, SD = 27.4, before HTP use M = 44.4%, SD = 17.0, after HTP use M = 22.7%, SD = 25.4; all results are displayed in Fig. 1).

**Figure 2:**
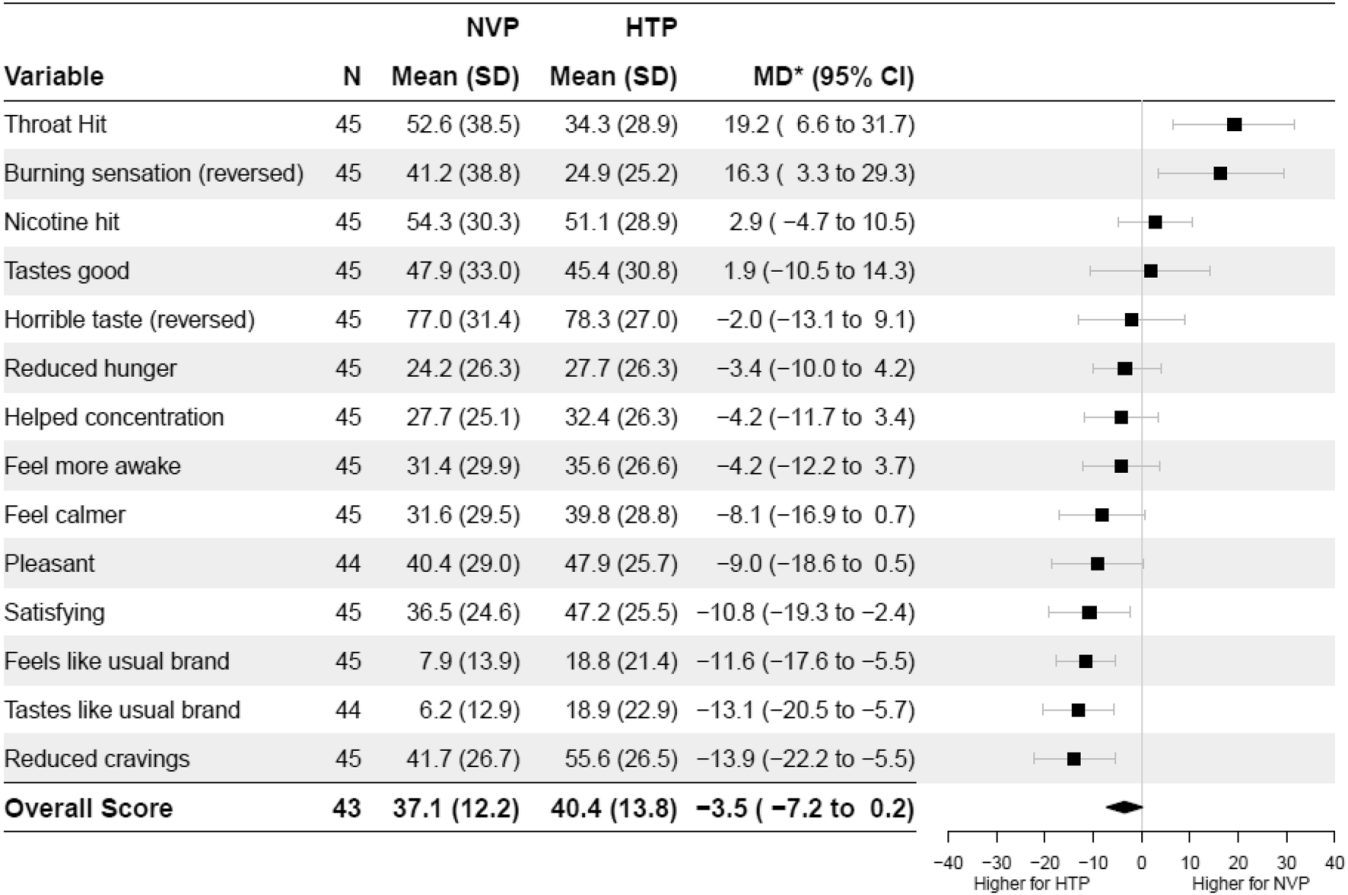
Acute positive effects after NVP and HTP use. NVP = nicotine vaping product, HTP = heated tobacco product, SD = standard deviation, MD* = mean within-person difference in outcome after NVP use minus HTP use with adjustment for period, CI = Confidence Intervals. Answer option a 10-point Likert scale, 1 = ‘not at all’ to 10 = ‘extremely’, normalised on 0% to 100% scale.

**Figure 3:**
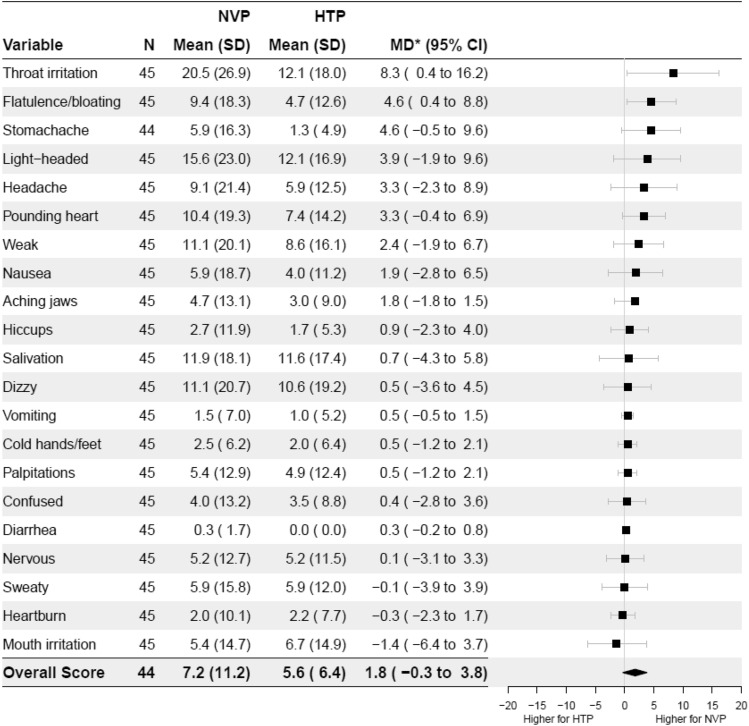
Adverse side-effects after NVP and HTP use. NVP = nicotine vaping product, HTP = heated tobacco product, SD = standard deviation, MD* = mean within-person difference in outcome after NVP use minus HTP use with adjustment for period, CI = Confidence Intervals. Answer option a 10-point Likert scale, 1 = ‘not at all’ to 10 = ‘extremely’, normalised on 0% to 100% scale.
  - Similarly, when looking at individual adverse side-effect scores, participants reported more throat irritation from the NVPs than the HTPs (Fig. 3). These differences in throat hit, burning sensation, and throat irritation were driven by the participants who used the Aspire PockeX NVP device; mean rating to these items were much higher for participants who used the Aspire PockeX than for Juul (burning cessation: Aspire PockeX mean (M) = 63.6%, Standard deviation (SD) = 35.2, Juul M = 13.3%, SD = 21.2; Aspire PocketX versus Juul MD = 50.2%, 95% CI 32.2, 68.3; throat hit: Aspire PockeX M = 68.9%, SD = 36.7, Juul M = 32.2%, SD = 30.6, Aspire PocketX versus Juul MD = 36.7%, 95% CI 16.0, 57.3; throat irritation: Aspire PockeX M = 33.8%, SD = 29.3, Juul M = 3.9%, SD = 8.3, Aspire PocketX versus Juul MD = 29.9%, 95% CI 16.2, 43.5).
(iii) Intention to switch to NVPs/HTPs
  The mean intention to switch was similar for both NVPs (M = 39.3%, SD = 27.8) and HTPs (M = 34.8%, SD = 27.5) after trying each product (MD = 4.0%, 95% CI − 5.7, 13.8).
(iv) Perceptions about NVPs/HTPs compared with cigarettes
  Figure 4 shows participants’ perceptions about NVPs and HTPs compared with cigarettes. When reporting on the perceived addictiveness of each product compared with cigarettes, two participants chose ‘don’t know’ for NVPs and five for HTPs. When excluding these participants, both products were perceived as having similar addictiveness (48.3% vs 44.4%, MD = 3.2%, 95% CI = − 5.9, 12.3). Participants reported that the HTP was more satisfying than the NVP (17.8% vs 30.6%; MD = – 13.2%, 95% CI − 20.3, − 6.1), though their scores indicated that both products were less satisfying compared with cigarettes. When reporting on the perceived safety of these products relative to cigarettes, seven chose ‘don’t know’ for NVPs compared with ten for HTPs. After excluding these participants, both products were generally judged to be safer than cigarettes, with little difference between HTPs and NVPs (33.6% vs 35.0%, MD = – 0.8%, 95% CI − 8.9, 7.4). Results remained unchanged in sensitivity analyses where participants who responded “don’t know” were included in the middle of the scales (see Supplementary Fig. 1).

**Figure 4:**
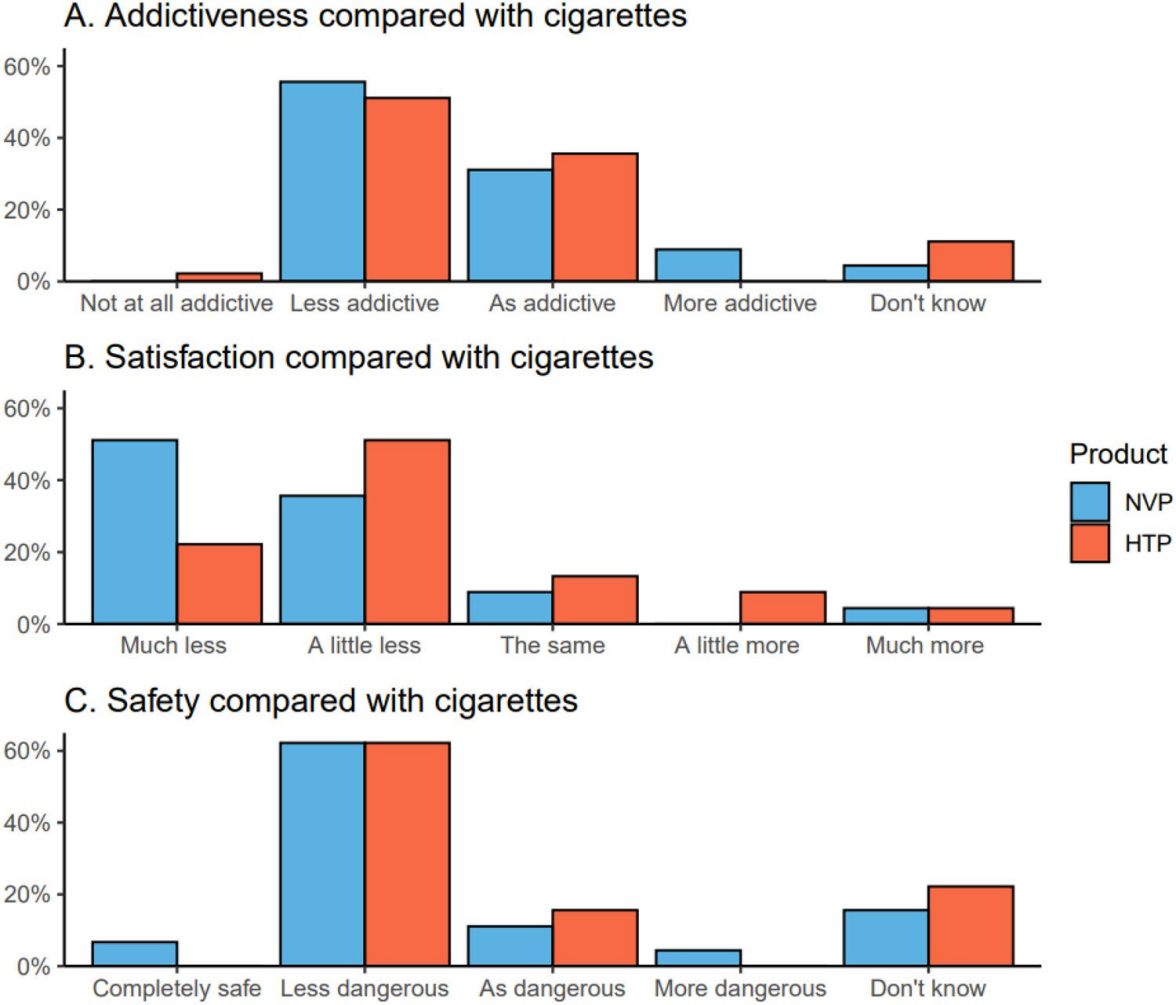
Perceptions about the addictiveness, satisfaction, and safety of NVPs and HTPs compared with cigarettes.
(v) Attitudes
  Participants had similar attitudes towards NVPs and HTPs after use (Supplementary Fig. 2). On average, participants reported that they would probably use both products indoors at home (60.0% vs 51.1%, MD = 8.9%, 95% CI − 2.0, 19.7), and outdoors (74.4% vs 68.3%, MD = 5.6%, 95% CI − 3.2, 14.4), but they would probably not use the products indoors at work and public spaces (36.7% vs 27.8%; MD = 8.8%, 95% CI − 0.3, 17.8). They responded that they might recommend the products to a friend who wanted to stop smoking (56.7% vs 54.0%, MD = 4.2%, 95% CI − 3.7, 12.1). Participants did not find the products embarrassing to use in the company of others (11.1% vs 10.6%, MD = 0.5%, 95% CI − 5.5, 6.4), and, on average, they reported that both products were somewhat helpful in enabling them to refrain from smoking (37.8% vs 43.3%, MD = – 5.7%, 95% CI − 14.6, 3.2) and somewhat pleasant to use (38.3% vs 40.9%; MD = – 3.5%, 95% CI − 14.9, 7.9).
(vi) Reasons that would motivate switching from cigarettes to NVPs/HTPs
  There was little difference between products in reasons motivating participants to switch. Generally, all but one reason were endorsed, with the most highly rated focusing on the product being ‘less harmful than tobacco cigarettes’, ‘satisfying’, ‘less harmful to other people around’, and helpful to ‘stop smoking’ and ‘to cut down smoking’ (Fig. 5).

**Figure 5:**
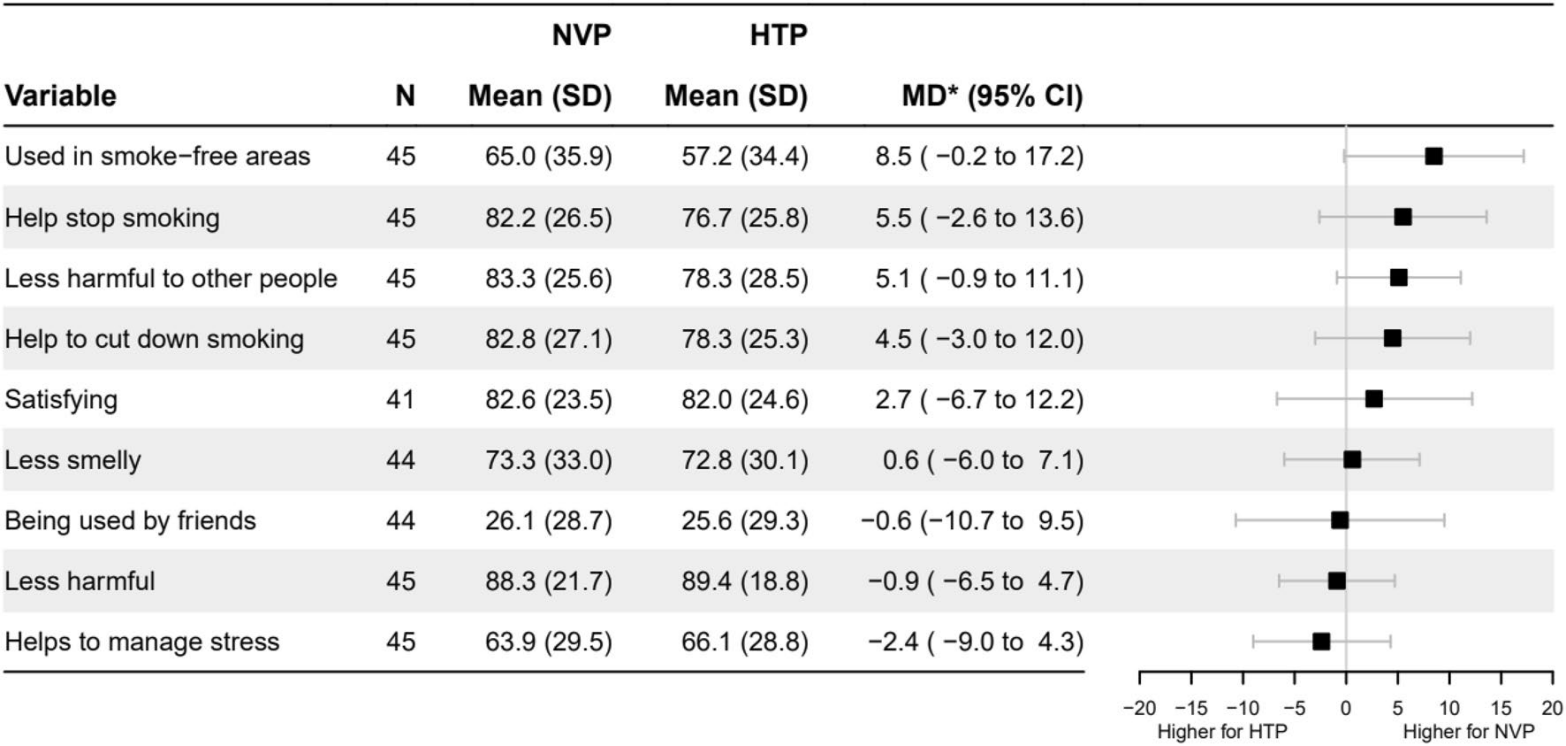
Reasons that would motivate switching from cigarettes to NVPs/HTPs. NVP = nicotine vaping product, HTP = heated tobacco product, SD = standard deviation, MD* = mean within-person difference in outcome after NVP use minus HTP use with adjustment for period, CI = Confidence Intervals. Normalised on 0% to 100% scale. Answer options on a 5-point Likert scale, 1 = ‘not at all’ to 5 = ‘extremely’, normalised on 0% to 100% scale.

## Discussion

In this cross-over randomized study of UK adult cigarette smokers naïve to NVPs and HTPs, we directly compared NVP and HTP short-term use in terms of cigarette-related withdrawal relief, acute positive effects and adverse side-effects, intention to switch and reasons for motivating smokers switching, as well as attitudes towards them and perceptions about them compared to cigarettes, and we observed little differences. Both products reduced cigarette cravings and withdrawal symptoms effectively and to a similar degree. Acute positive effects and adverse side-effects were comparable, if marginally favouring HTPs. Relative to cigarettes HTPs were perceived as more satisfying than NVPs but both products were seen as addictive as each other compared with cigarettes. Lastly, intention to switch to either product did not differ.

Consistent with previous findings demonstrating reliable nicotine delivery after acute administration using NVP and HTP (e.g.,^31,32,34^), our results indicated that urge to smoke and nicotine-related withdrawal symptoms were reduced after each product use. Since HTPs perform similarly to NVPs in cigarette-related withdrawal relief and available evidence indicates that NVPs are effective for cigarette smoking reduction and cessation (i.e.,^13,17,20^), this suggest that HTPs may prove similarly effective in smoking cessation as NVPs.

Levels of acute positive effects were close to the middle values of the scales, and slightly higher after HTP use compared to after NVP use. Participants felt that the HTP had reduced their craving for nicotine, it was satisfying, and it felt like their usual cigarette brand. Unlike NVPs, HTPs contain tobacco leaf, and their flavour may more closely resemble cigarette smoke^51^, which is also reflected in our findings. Similar to previous research (e.g.^32,36^) adverse side-effects after each product use were minimal, though slightly higher after NVP use. However, this may be a function of the specific NVP used as items related to throat irritation and burning cessation were particularly associated with Aspire PocketX device and not Juul use. Juul is among the first NVP devices to use e-liquids that contain nicotine salts rather than freebase nicotine. Nicotine salt formulations have a pH that is more similar to the extravascular fluid in the lung than non-protonated nicotine. This allows users to vape much higher concentrations of nicotine without experiencing irritation to the throat^50,52^, which may explain the different experience between the two NVP products.

The minimal adverse side-effects, the middling acute positive effects and the favourable attitudes reported after NVP and HTP use may indicate these products are viable cigarette substitutes that could promote switching. However, when participants were asked directly their intention to switch to these products, 46.7% and 42.2% replied that this was somewhat unlikely for NVP and HTP, respectively. One reason for this is that even the HTP, which was considered more satisfying than the NVP, was still perceived as less satisfying compared with cigarettes. It could also be attributed to the fact that motivation to quit smoking assessed before product use was moderate, and this acute experience of NVP/HTP use was not enough to change participants’ intention to substitute smoking with another nicotine delivery device. Additionally, our sample included adult cigarette smokers naïve to NVPs and HTPs, which may further suggest that these people purposively stayed away from trying to use these products and thus might be harder to persuade to switch. Interestingly, a few participants who reported no intention to quit smoking in the next 3 months (based on the MTSS scale), reported that they were somewhat likely/very likely switching to NVPs and HTPs (22.2% and 20.0% respectively), which may indicate that these products could substitute cigarette smoking in smokers unmotivated to quit and contribute to harm reduction. Similar to previous research among cigarette smokers, we found that the most important reasons for switching to NVPs/HTPs were satisfaction, to reduce harm to themselves and others, to reduce smoking, and to help them stop smoking^53,54^. Current prevalence of adult NVP and HTP use in England is 9.3% and 0.2% respectively, though our sample showed similar levels of intention to switch to either product after trying them. This might be due to the fact that only NVPs are promoted as an effective way of quitting cigarette smoking in UK and that NVPs have been more widely available for a longer period of time than HTPs. Additionally, poor marketing may have also contributed to the poor diffusion of HTPs in England.

Finally, views on the safety of these novel products were somewhat out of step with the evidence. NVPs generally expose users to much lower levels of carcinogens and toxicants than cigarettes^13,14^, and have been promoted as less harmful alternatives to cigarette smoking for smokers struggling to quit in UK^9^. By contrast, there is insufficient independent evidence to draw strong conclusions about the safety of HTP^9^, although newer studies are consistent with reduced harm^55,56^, and recently in the US, the FDA has authorized the legal commercialization of HTPs, affirming “that marketing of these products is appropriate for the protection of public health” based on the FDA’s scientific evaluation of reduced consumers’ exposure to harmful chemicals when completely switching away from conventional cigarettes^57^. Yet, when both products were compared to cigarettes, most respondents perceived them to be equally as harmful as each other, and a fair number considered them to be as harmful as, or more harmful than, cigarettes. Similar findings regarding NVPs safety were observed in a cohort study in Canada^58^, and it has been suggested that after the US outbreak of vaping-associated lung injury^59^, views on NVPs among smokers deteriorated and many smokers perceiving NVPs at least as harmful to health as cigarettes^60,61^. Likewise, studies examining perceptions of harmfulness of HTPs compared to cigarettes suggest that a fair number of smokers considered them to be equally as harmful as cigarettes or they do not know^53,62^.

All results obtained should be seen in the light of the following limitations. First, the results and their interpretations are based on a small convenience sample of London-based adults. Though considering the cross-over design of the study, the sample is equivalent to having at least double the sample of a parallel-group randomised trial. Nonetheless, the sample is unlikely to be representative for the average regular smoker. Yet, many of our findings are comparable to other studies (i.e.^32,34,54,58^) suggesting some, albeit limited, generalisability to other contexts. Additionally, both NVPs and HTPs are available in UK, while most of the available data on HTPs come from Japan where NVPs are not available. Second, participants only received a brief explanation on how to use the two products, which were new for them, and they got no more than five minutes to use each product, while it is well established that people may need to experiment for a couple of days with these products to learn to use them effectively^25^. Though first impressions (i.e., craving reduction, satisfaction) also count and may contribute to continue use of a product (or not), so it is important to capture information on effects of acute use in terms of likely uptake/continued use. Third, for the NVP, we used only two different devices and for the HTP just one specific type. Whereas the offer of the different HTP is rather limited, a plethora of NVPs and of flavours of e-liquids with various nicotine concentrations are available, which may well differ with respect to the effect of the outcomes studied here. Fourth, we used validated measures for some of the outcomes (i.e.,^46,47^), while for others we used measures based on previous research (i.e.,^33,48^). Fifth, in this study we used two NVP devices (Juul and Aspire PockeX,) and one HTP device (IQOS). Thus, these results may not generalise to other types of NVP or HTP. Sixth, the results need to be treated as preliminary and that studies with longer user and follow-up are needed to confirm our findings.

To conclude, comparison of acute use of NVP versus HTP in a sample of UK smokers naïve to these products suggests that HTPs are perceived as more satisfying than NVPs, though still less satisfying than cigarettes. Few differences were observed between the two products for other outcomes. Both products reduced cigarettes cravings and were seen as similarly addictive to each other when compared to cigarettes. The perceived adverse side-effects after using each product were minimal, apart from throat irritation after Aspire PockeX NVP use. Acute positive effects were modest and attitudes towards both products were favourable, though intention to switch to either product was relatively low. Nonetheless, given the comparable psychopharmacological profile and perceived acceptability of both products, taken together with the now established effectiveness of NVP for smoking cessation, this would suggest that HTP may play a useful role in combustible tobacco harm reduction.

### Supplementary Information

Supplementary Information.

## Data Availability

The dataset used and analysed during the current study are available from the corresponding author (Dimitra Kale) on reasonable request.
