# Supplementary material for: Examining acute psychopharmacological effects of nicotine vaping versus heated tobacco products in a randomised crossover study of product naïve adult smokers
Source: Sci Rep. 2023 Dec 19;13:22676. doi: 10.1038/s41598-023-49602-3 (PMC10730620; doi:10.1038/s41598-023-49602-3)
Supplement: Supplementary file 1 — Supplementary Information. [file 41598_2023_49602_MOESM1_ESM.docx]

**Supplementary Materials**

Supplementary table 1. Description of outcomes

| Outcome | Question assessed | Answer options |
| --- | --- | --- |
| **i. Withdrawal relief** | | |
| Momentarily cigarette craving strength (MPPS) | How strong are the urges to smoke a cigarette right now? | 1. No urges  2. Slight  3. Moderate  4. Strong  5. Very strong  6. Extremely strong |
| Mood and physical symptoms related to withdrawal symptoms | Please show for each of the items below how you are feeling right now. | |
|  | Irritable | 1. Not at all  2. Slightly  3. Somewhat  4. Very  5. Extremely |
|  | Restless |  |
|  | Depressed |  |
|  | Anxious |  |
|  | poor concentration |  |
|  | Hungry |  |
| **ii. Product satisfaction** | | |
| Acute positive effects | Please indicate the extent to which you agree with each of the following statements based on how you feel now. | |
|  | I have felt a definite throat hit from the NVP/HTP | 1. Not at all  2.  3.  4.  5.  6.  7.  8.  9.  10. Extremely |
|  | The NVP/HTP is pleasant |  |
|  | The NVP/HTP is satisfying |  |
|  | The NVP/HTP tastes good |  |
|  | The NVP/HTP has reduced my craving for nicotine |  |
|  | The NVP/HTP has helped my concentration |  |
|  | The NVP/HTP has made me feel calmer |  |
|  | The NVP/HTP has made me feel more awake |  |
|  | The NVP/HTP has reduced my hunger |  |
|  | The NVP/HTP tastes like my usual brand/model |  |
|  | The NVP/HTP feels like using my usual brand/model |  |
|  | I have felt a definite nicotine hit from the NVP/HTP |  |
|  | I have felt a burning sensation when using the NVP/HTP (reversed) |  |
|  | I have experienced a horrible taste from using the NVP/HTP (reversed) |  |
| Adverse side-effects | Please indicate the extent to which you feel each of the following right now. | |
|  | Confused | 1. Not at all  2.  3.  4.  5.  6.  7.  8.  9.  10. Extremely |
|  | Dizzy |  |
|  | Headache |  |
|  | Pounding heart |  |
|  | Light-headed |  |
|  | Nausea/feeling sick |  |
|  | Nervous |  |
|  | Salivation |  |
|  | Sweaty |  |
|  | Weak |  |
|  | Mouth irritation |  |
|  | Throat irritation |  |
|  | Aching jaws |  |
|  | Vomiting |  |
|  | Flatulence/bloating |  |
|  | Stomach-ache |  |
|  | Heartburn |  |
|  | Diarrhoea |  |
|  | Hiccups |  |
|  | Cold hands/feet |  |
|  | Palpitations |  |
| **iii. Intention to switch to NVP/HTP** | | |
|  | How likely are you to switch from cigarettes to NVP/HTP? | 0. Very unlikely  1. Somewhat unlikely  2. Somewhat likely  3. Very likely |
| **iv. Perceptions about NVP/HTP compared with cigarettes** | | |
|  | Compared with cigarettes, do you think that the NVP/HTP is…? | 1. Completely safe  2. Less dangerous than cigarettes  3. As dangerous as cigarettes  4. More dangerous than cigarettes  Don’t know |
|  | Compared with cigarettes, do you think that the NVP/HTP is…? | 1. Not at all addictive  2. Less addictive than cigarettes  3. As addictive as cigarettes  4. More addictive than cigarettes  Don’t know |
|  | Compared to tobacco cigarettes how satisfying is the NVP/HTP? | 1. Much less than usual  2. A little less than usual  3. The same as usual  4. A little more than usual  5. Much more than usual |
| **v. Attitudes** | | |
|  | How helpful do you find the NVP/HTP in enabling you to keep from smoking? | 1. Not at all helpful  2. Slightly helpful  3. Somewhat helpful  4. Very helpful  5. Extremely helpful |
|  | How pleasant is the NVP/HTP to use? | 1. Not at all pleasant  2. Slightly pleasant  3. Somewhat pleasant  4. Very pleasant  5. Extremely pleasant |
|  | How embarrassing is the NVP/HTP to use in the company of others? | 1. Not at all embarrassing  2. Slightly embarrassing  3. Somewhat embarrassing  4. Very embarrassing  5. Extremely embarrassing |
|  | Would you recommend the NVP/HTP to a friend who wanted to stop smoking? | 1. Definitely not  2. Probably not  3. Maybe  4. Probably  5. Definitely |
|  | Would you use the NVP/HTP outdoor? |  |
|  | Would you use the NVP/HTP indoor at home? |  |
|  | Would you use the NVP/HTP indoor at work/public spaces (i.e., a restaurant)? |  |
| **vi. Reasons that would motivate switching from cigarettes to NVPs/HTPs** | | |
|  | How important are the following reasons in motivating you to switch from cigarettes to the NVP/HTP? | |
|  | If the NVP/HTP is satisfying | 1. Not at all  2.  3.  4.  5. Extremely |
|  | If the NVP/HTP is less smelly (than tobacco cigarettes) |  |
|  | If the NVP/HTP is being used by your friend(s) |  |
|  | If the NVP/HTP helps to manage stress |  |
|  | If the NVP/HTP is less harmful than tobacco cigarettes |  |
|  | If the NVP/HTP is less harmful to other people around you |  |
|  | If the NVP/HTP can be used in smoke-free areas |  |
|  | If the NVP/HTP can help to stop smoking |  |
|  | If the NVP/HTP can help to cut down smoking |  |

Supplementary figure 1. Perceptions of NVPs/HTPs compared to cigarettes after NVP/HTP use


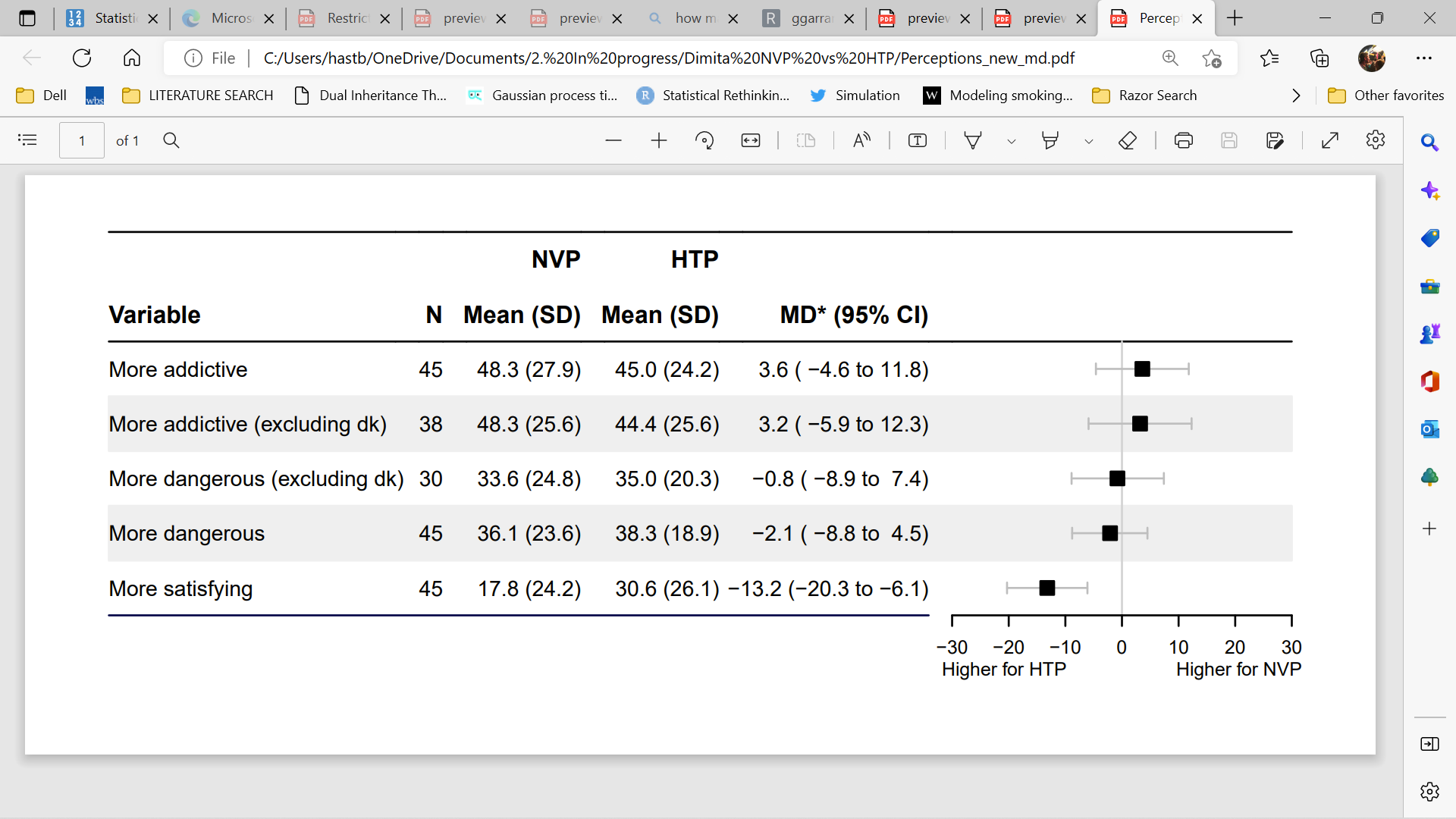


NVP=nicotine vaping product, HTP=heated tobacco product, SD=standard deviation, MD*=mean within-person difference in outcome after NVP use minus HTP use with adjustment for period, CI=Confidence Intervals, dk=don’t know

Supplementary figure 2. Attitudes towards NVPs/HTPs after use


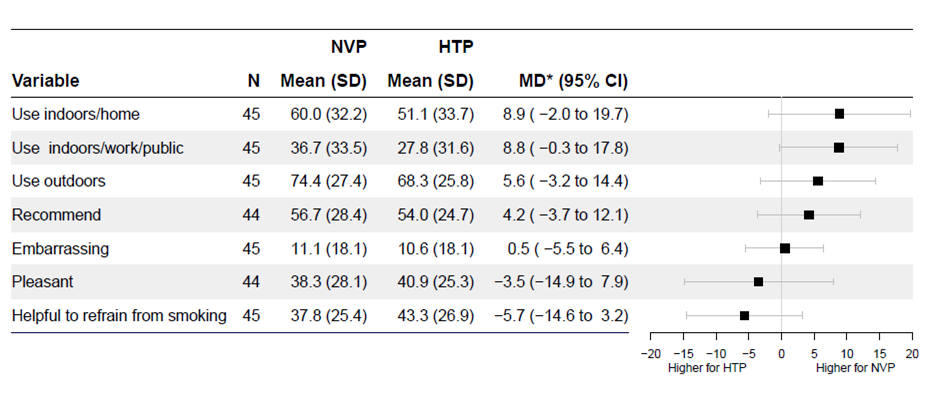


NVP=nicotine vaping product, HTP=heated tobacco product, SD=standard deviation, MD*=mean within-person difference in outcome after NVP use minus HTP use with adjustment for period, CI=Confidence Intervals.
